# Cardiac surgery in a patient with retroperitoneal fibrosis and heart valvulopathy, both due to pergolide medication for Parkinson's disease

**DOI:** 10.1186/1749-8090-4-65

**Published:** 2009-11-13

**Authors:** Efstratios E Apostolakis, Nikolaos G Baikoussis, Dimitrios Tselikos, Ioanna Koniari, Christos Prokakis, Eleftherios Fokaeas, Menelaos Karanikolas

**Affiliations:** 1 Department of Cardiothoracic Surgery, University of Patras, School of Medicine. Patras, Greece; 2 Department of Urology, University of Patras, School of Medicine, Patras, Greece; 3 Department of Anaesthesiology and Critical Care Medicine, University of Patras, School of Medicine. Patras, Greece

## Abstract

Retroperitoneal fibrosis is best described as a chronic inflammatory process which may be idiopathic, but can rarely be brought about by medications, such as pergolide, used for treating Parkinson's disease. Pergolide can produce a fibrotic process in heart valves, resulting in valve insufficiency in up to 25% of cases. Herein we describe the case of a 68-year-old man who received pergolide for 2 years for Parkinson's disease. The patient developed retroperitoneal fibrosis resulting in renal failure from ureteral obstruction necessitating ureteral stenting, as well as significant aortic and mitral valve insufficiency. He successfully underwent surgery for combined aortic valve, mitral valve and ascending aorta replacement because of severe valve insufficiency and dilated (d = 5.8 cm) ascending aorta. Retroperitoneal fibrosis improved with pergolide cessation and corticosteroid treatment. This is the second case reported in the literature, of a patient who had double valve and ascending aorta replacement surgery because he suffered from this rare but serious adverse effect of dopamine agonists used for managing Parkinson's disease.

## Introduction

Retroperitoneal fibrosis (RPF) describes a chronic inflammatory process of the retroperitoneum, with eventual fibrosis and entrapment of the ureters and other retroperitoneal organs, which can produce obstructive uropathy and renal failure [1,2]. Rarely, is RPF related to drugs overt autoimmune disease and chronic infection, such as tuberculosis [1,3]. In fact, retroperitoneal or pleural fibrosis, the so called "serosal fibrosis" secondary to pergolide has been reported by many authors [4,5]. Apart from the above mentioned serosal fibrosis, another consequence of ergot dopamine agonists, such as pergolide, is heart valve regurgitation. Van Camp G et al [4] reported the development of moderate-to-severe heart-valve regurgitation in 15 of 78 patients treated with pergolide for Parkinson's disease. The changes mediated by the 5-HT_2B _agonist are closely connected to the serotoninergic receptors expressed on cardiac valvular fibroblasts [6,7]. In fact, pergolide and cabergoline have high "affinity" for the 5-HT_2B _serotonin receptors, which are expressed in heart valves and might mediate mitogenesis and, in turn, the proliferation of fibroblasts. The latter process causes fibrotic changes such as thickening, retraction, and stiffening of valves, which result in incomplete leaflet closure and clinically significant regurgitation [7]. Fortunately, heart valve replacement will only be necessary in a few of these patients.

### Our Case

**Figure 1:**
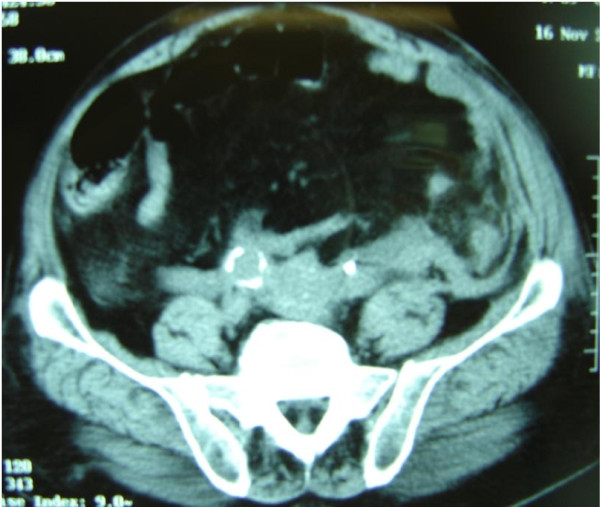
**Preoperative CT scan showing diffuse retroperitoneal fibrosis**.

A 68-year-old man was admitted with acute pulmonary edema and ever worsening symptomatology throughout the past 6 months. His symptoms included exercise-induced dyspnea and paroxysmal nocturnal dyspea (NYHA III). From his past medical history we noted Parkinson's disease diagnosed three years ago. Pergolide treatment (1 mg twice a day) ameliorated the tremor, but the patient developed oliguria and deterioration of renal function 18 months later. CT of the abdomen showed diffuse retroperitoneal fibrotic tissue with bilateral kidney and ureter compression, resulting in right kidney hydronephrosis, and a 7 cm-long dense tissue mass in the retroperitoneal space, below the L 5 vertebra, near the great vessels. Two endo-ureteral stents were inserted and restored patency of both ureters. Renal function temporarily improved, but deteriorated again with worsening fibrosis (figure 1). Echocardiography and Doppler examination revealed moderate (2+/4+) aortic valve regurgitation with thickening and calcification of the aortic valve leaflets, mitral valve insufficiency (1+/4+) with similar lesions, and dilatation of the ascending aorta with a diameter of 5 cm. Left ventricular function was affected, with injection fraction (EF) of 50%. Repeated observation over the ensuing 18 months revealed gradual deterioration of aortic and mitral insufficiency and LV function. Medical management, including therapy with diuretics (oral furosemide 80 mg/24 h) temporarily controlled his symptoms. However, in the following 6 months renal function deteriorated dramatically, to the point where the patient required haemodialysis 3 times per week. After an emergency admission to our hospital for acute dyspnea, repeat echocardiography revealed severe (3+/4+) aortic and mitral valve insufficiency, together with further deterioration of left ventricular function (EF = 40%), whereas coronary angiography revealed normal coronary arteries.

**Figure 2:**
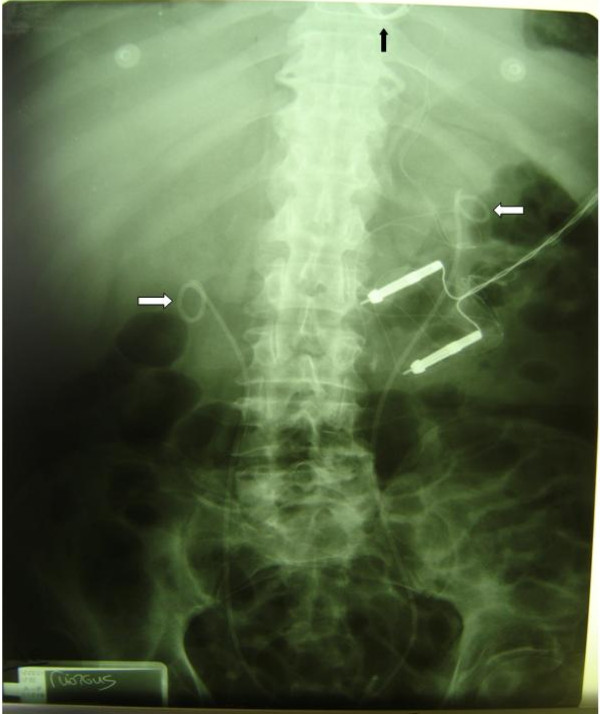
**An x-ray performed during the early postoperative period**. The ureteral catheters are showing (white arrows) while the annulus of the mechanical valves (black arrow) and the wires of the epicardial pace maker are also seen.

The patient underwent elective cardiac surgery, for double (aortic and mitral) valve replacement combined with ascending aorta replacement. Haemodialysis was performed in the afternoon before the scheduled operation and every other day postoperatively. The operation was conducted under cardiopulmonary bypass, systemic hypothermia at 28°C and meticulous myocardial protection with combination of intermittent antegrade and retrograde cardioplegia. The patient had aortic (mechanical Sorin Pericarbon 21 mm), mitral (mechanical Sorin Pericarbon 27 mm) and ascending aorta (woven Dacron graft of 30 mm) replacement. The native valve cusps were thickened and had dense, diffuse fibrosis and some calcification. The histopathologic examination revealed diffuse excessive fibrosis, local hyelinosis and dystrophic calcifications. The early postoperative course was uneventful, and the patient only required hemodynamic support with low doses of adrenaline (3-6 μ/Kg/min) and "renal dose" dopamine (6 μg/Kg/min) (figure 2). The patient was discharged from the hospital on the 16^th ^postoperative day in good condition. Pergolide discontinuation and cortisol treatment resulted in gradual improvement of retroperitoneal fibrosis, with significant improvement of renal function (urea = 80 mg % and creatinine = 2 mMol/L). Today, 42 months after this complex cardiac operation, the patient is in good health and does not need haemodialysis any longer.

## Discussion

Pergolide, a drug used for treating Parkinson's disease, can cause retroperitoneal fibrosis, as well as a dose-dependent heart valve fibrotic process, leading to severe valve insufficiency after two to three years of treatment [5-7]. There are no large series or case reports of patients undergoing cardiac surgery for double valvulopathy due to pergolide. Zanettini et al [6] examined the risk of heart valve degeneration and severity of valve disease by comparing 64 patients taking pergolide with 49 patients taking cabergoline, 42 patients taking a non-ergot derivative, and 90 control patients, and showed that the frequency of clinically important regurgitation in any cardiac valve was significantly higher in patients taking pergolide (23.4%) or cabergoline (28.6%), compared to patients taking non-ergot dopamine agonists (0%) or controls (5.6%). New evidence from population studies comparing patients with Parkinson's disease and non-parkinsonian controls suggests that the risk of substantial valve regurgitation is 5-6 times higher in patients with Parkinson's disease treated with cabergoline, and documents the occurrence of cardiac valvulopathy in patients treated with pergolide at doses around 3 mg/day or more [5]. A similar study from Japan [7] reported a significantly (p < 0·05) increased risk of echocardiographically significant valvular regurgitation in patients taking cabergoline but not in those receiving pergolide. The reasons for the observed lower incidence of valve regurgitation in the Japanese study in comparison to Europeans studies is unclear and may be related to the lower pergolide doses used in Asian patients. There are only a few reported cases of patients who had surgery for cardiac disease acquired due to medications given for treatment of Parkinson's disease: **A) **by Zanettini et al [6], a 69-year-old man taking pergolide underwent mitral-valve and aortic-valve replacement for severe mitral regurgitation and moderate aortic regurgitation. The surgeon described the mitral and aortic valve leaflets in this patient as diffusely thickened and retracted. **B) **By Camp G, et al [8], a 73-year old female taking pergolide presented with a new holosystolic murmur, and required aortic valve replacement. In conclusion, we suggest that every patient taking pergolide for Parkinson's disease should be subjected to ECHO examination every six months, for heart valve function assessment.

## Consent

Written informed consent was obtained from the patient for publication of this case report and accompanying images. A copy of the written consent is available for review by the journal Editor-in-Chief.

## Competing interests

The authors declare that they have no competing interests.

## Authors' contributions

All authors: 1. have made substantial contributions to conception and design, or acquisition of data, or analysis and interpretation of data; 2. have been involved in drafting the manuscript or revisiting it critically for important intellectual content; 3. have given final approval of the version to be published.
